# Single-cell imaging of inflammatory caspase dimerization reveals differential recruitment to inflammasomes

**DOI:** 10.1038/cddis.2015.186

**Published:** 2015-07-09

**Authors:** M G Sanders, M J Parsons, A G A Howard, J Liu, S R Fassio, J A Martinez, L Bouchier-Hayes

**Affiliations:** 1 Department of Pediatrics-Hematology, Baylor College of Medicine, Houston, TX, USA; 2 Center for Cell and Gene Therapy, Baylor College of Medicine, Houston, TX, USA; 3 Department of Biochemistry and Cell Biology, Rice University, Houston, TX, USA; 4 Department of Immunity, Inflammation and Disease, National Institute of Environmental Health Sciences, 111 TW Alexander Drive, Research Triangle Park, NC, USA; 5 Department of Molecular and Cellular Biology, Baylor College of Medicine, Houston, TX, USA

## Abstract

The human inflammatory caspases, including caspase-1, -4, -5 and -12, are considered as key regulators of innate immunity protecting from sepsis and numerous inflammatory diseases. Caspase-1 is activated by proximity-induced dimerization following recruitment to inflammasomes but the roles of the remaining inflammatory caspases in inflammasome assembly are unclear. Here, we use caspase bimolecular fluorescence complementation to visualize the assembly of inflammasomes and dimerization of inflammatory caspases in single cells. We observed caspase-1 dimerization induced by the coexpression of a range of inflammasome proteins and by lipospolysaccharide (LPS) treatment in primary macrophages. Caspase-4 and -5 were only dimerized by select inflammasome proteins, whereas caspase-12 dimerization was not detected by any investigated treatment. Strikingly, we determined that certain inflammasome proteins could induce heterodimerization of caspase-1 with caspase-4 or -5. Caspase-5 homodimerization and caspase-1/-5 heterodimerization was also detected in LPS-primed primary macrophages in response to cholera toxin subunit B. The subcellular localization and organization of the inflammasome complexes varied markedly depending on the upstream trigger and on which caspase or combination of caspases were recruited. Three-dimensional imaging of the ASC (apoptosis-associated speck-like protein containing a caspase recruitment domain)/caspase-1 complexes revealed a large spherical complex of ASC with caspase-1 dimerized on the outer surface. In contrast, NALP1 (NACHT leucine-rich repeat protein 1)/caspase-1 complexes formed large filamentous structures. These results argue that caspase-1, -4 or -5 can be recruited to inflammasomes under specific circumstances, often leading to distinctly organized and localized complexes that may impact the functions of these proteases.

Correct assembly and regulation of inflammasomes is critical for mediating inflammation and preventing uncontrolled inflammation under infectious and sterile conditions. These supramolecular structures converge on the activation of caspase-1. Upon activation, caspase-1 cleaves the proinflammatory cytokines interleukin1*β* (IL-1*β*) and IL-18 to their active mature forms,^1, 2^ which are then released from the cell to direct the immediate removal of pathogens.

Caspase-1 is activated by proximity-induced dimerization upon recruitment to inflammasomes, which are multiprotein signaling complexes that act as activation platforms.^3^ Many distinct inflammasomes exist, and each inflammasome includes a sensor protein (e.g., NALP1 (NACHT leucine-rich repeat protein 1)/NLRP1 (NOD-like receptor protein 1), NALP3/NLRP3, AIM2 (absent in melanoma 2) or IPAF (ICE protease-activating factor)/NLRC4 (NLR family CARD domain-containing protein 4)), which is activated by specific proinflammatory molecules. These include pathogen-derived stimuli, known as pathogen-associated molecular patterns (PAMPs), or non-pathogenic inflammatory stimuli, known as damage-associated molecular patterns.^4^ Inflammasome assembly is governed by a series of homotypic interactions, which are mediated by specific protein:protein interaction domains, such as the pyrin domain (PYD) and the caspase recruitment domain (CARD).^5, 6^ For example, NALP1 and NALP3 both contain a PYD at their C terminus that binds to the PYD in the adaptor protein, ASC (apoptosis-associated speck-like protein containing a CARD).^7, 8^ ASC also contains a CARD,^9^ which binds to the CARD in the prodomain of caspase-1 (C1-Pro), resulting in caspase-1 dimerization and activation.^10^ Inflammasomes can also be ASC-independent, such as IPAF, which interacts directly with caspase-1.^11^ Interestingly, ASC can enhance IPAF-induced caspase-1 activation,^12, 13^ which indicates that more complex interactions between these proteins may exist.

Caspase-1 is one of the inflammatory caspases, including the human caspases (caspase-1, -4, -5 and -12) and murine caspase-11.^14^ The roles of caspase-4, -5, and -12 in caspase-1 activation and inflammasome pathways are not clear. Full-length caspase-12, which is only expressed by ~20% of people of African descent, can inhibit caspase-1 activity.^15^ Most of all human populations express the short form of caspase-12 (caspase-12 S or C12S), which arose from a point mutation leading to a premature stop codon just after the prodomain. This truncated form of caspase-12 is associated with increased resistance to sepsis,^15^ indicating that caspase-12 has an important role in inflammation. Caspase-11 mediates caspase-1 activation in response to *Escherichia coli* and *Citrobacter rodentium* in mice.^16^ Caspase-11 also triggers an inflammatory form of cell death, known as pyroptosis, independent of caspase-1, ASC and NALP3. This is known as the noncanonical inflammasome pathway.^16^ Humans do not express caspase-11 and express caspase-4 and -5 instead. Recent evidence indicates that caspase-4 and -5 act as direct intracellular sensors for lipopolysaccharide (LPS) to induce pyroptosis, independent of any additional inflammasome proteins.^17^ However, this does not rule out the possibility that caspase-4 and -5 can be recruited to inflammasomes under certain circumstances.

The inflammatory caspases are considered to be initiator caspases, based on structural similarities between them and caspase-2, -8 and -9.^18^ We previously reported the use of caspase bimolecular fluorescence complementation (BiFC) to measure induced proximity of the initiator caspase, caspase-2.^19^ We adapted BiFC, where non-fluorescent fragments of the yellow fluorescent protein, Venus (‘split Venus'), can associate to reform the fluorescent complex when fused to interacting proteins. When caspase-2 was fused to each half of split Venus, the recruitment of caspase-2 to its activation platform and the subsequent induced proximity resulted in association of the two Venus halves. This resulted in an increase in fluorescence that represents caspase dimerization. Our caspase BiFC method facilitates specific analysis of caspase interactions at the level of the activation platform. Importantly, caspase BiFC can reveal the structural organization and localization of activation platforms in living cells.

Here, we extend the caspase BiFC approach to interrogate the inflammatory caspases. We show that there are considerable differences in the organization and distribution of different inflammasomes depending on the upstream signals and on which inflammatory caspase is recruited. Our studies reveal unexpected heterodimerization interactions between caspase-1 and additional inflammatory caspases, presenting a new outcome of inflammasome assembly.

## Results

### Reconstitution of inflammasomes in cells induces inflammatory caspase BiFC

**Figure 1:**
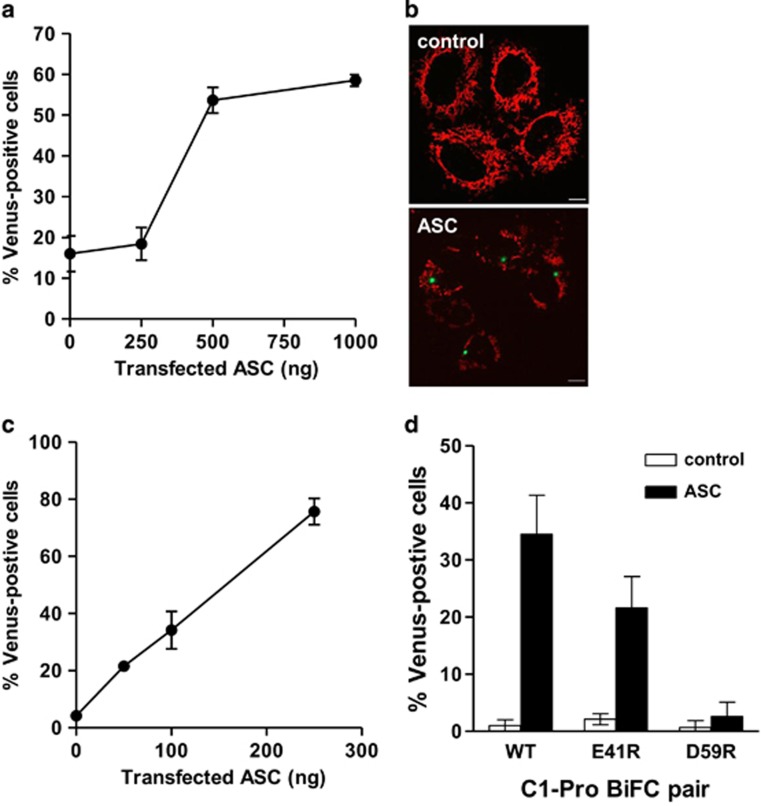
ASC induces caspase-1 BiFC. (**a**) HeLa cells were transiently transfected with C1-Pro VC (10 ng) and C1-Pro VN (10 ng) along with the indicated amounts of an expression plasmid encoding ASC. All wells also received dsRed-mito (10 ng) as a reporter for transfection. All cells were treated with qVD-OPH (5 *μ*M) to prevent cell death. At 48 h after transfection, the percentage dsRed-mito-positive cells that were Venus-positive was determined from a minimum of 300 cells per well. Results represent triplicate counts with error bars representing S.D. and are representative of at least three independent experiments. (**b**) Representative confocal images of cells from (**a**) are shown, showing mitochondria (red) and caspase-1 BiFC (green). Scale bars represent 10 *μ*m. (**c**) MCF-7 cells were transiently transfected with C1-Pro VC (10 ng) and C1-Pro VN (10 ng) along with the indicated amounts of an expression plasmid encoding ASC and dsRed-mito (10 ng). Cells were treated and assessed as in (**a**). (**d**) MCF-7 cells were transiently transfected with C1-Pro VC (10 ng) and C1-Pro VN (10 ng), the E41R mutant C1-Pro Venus pair (10ng of each) or the D59R mutant C1-Pro Venus pair (10ng of each) along with dsRed-mito (10 ng). Cells were treated and assessed as in (**a**)

We used the BiFC assay to study the recruitment of caspase-1 to its activation platforms and its subsequent dimerization. We fused the C1-Pro (aa 1–102) to each of the split Venus fragments, Venus C (VC; aa 155–239) and Venus N (VN; aa 1–173). We used the C1-Pro because it contains the CARD that interacts with inflammasomes but it is not catalytically active. Thus, expression of the BiFC constructs does not add any enzymatic activity that would contribute to downstream effects including cell death, facilitating analysis of dimerization of the caspase in response to specific upstream triggers. We transiently expressed the C1-Pro BiFC pair in HeLa cells and investigated their ability to undergo BiFC upon coexpression of the caspase-1 adaptor protein ASC. Upon increasing ASC expression, we observed a robust increase in the percentage of Venus-positive cells, indicating amplified induction of BiFC of the C1-Pro pair (Figure 1a). ASC-induced C1-Pro BiFC appeared as a single fluorescent punctum in each cell (Figure 1b). We observed similar results in the MCF-7 cell line (Figure 1c). MCF-7 cells do not express detectable levels of caspase-1, -4 or -5 (Supplementary Figure S1 and Lin *et al.*^20^). Therefore, we used this cell line for our next set of experiments to avoid potential interference from the endogenous inflammatory caspases.

Mutation of the CARD of caspase-1 at E41 or D59 has been shown to disrupt binding between caspase-1 and ASC.^21^ To control for specificity of the caspase-1 BiFC pair, we mutated these residues in the C1-Pro BiFC constructs. Although the E41R mutant C1-Pro BiFC pair showed slightly reduced levels of BiFC in response to ASC expression, the D59R mutation completely abrogated ASC-induced C1-Pro BiFC (Figure 1d). Thus, ASC-induced dimerization of caspase-1, as measured by BiFC, is dependent on the binding of caspase-1 to ASC.

**Figure 2:**
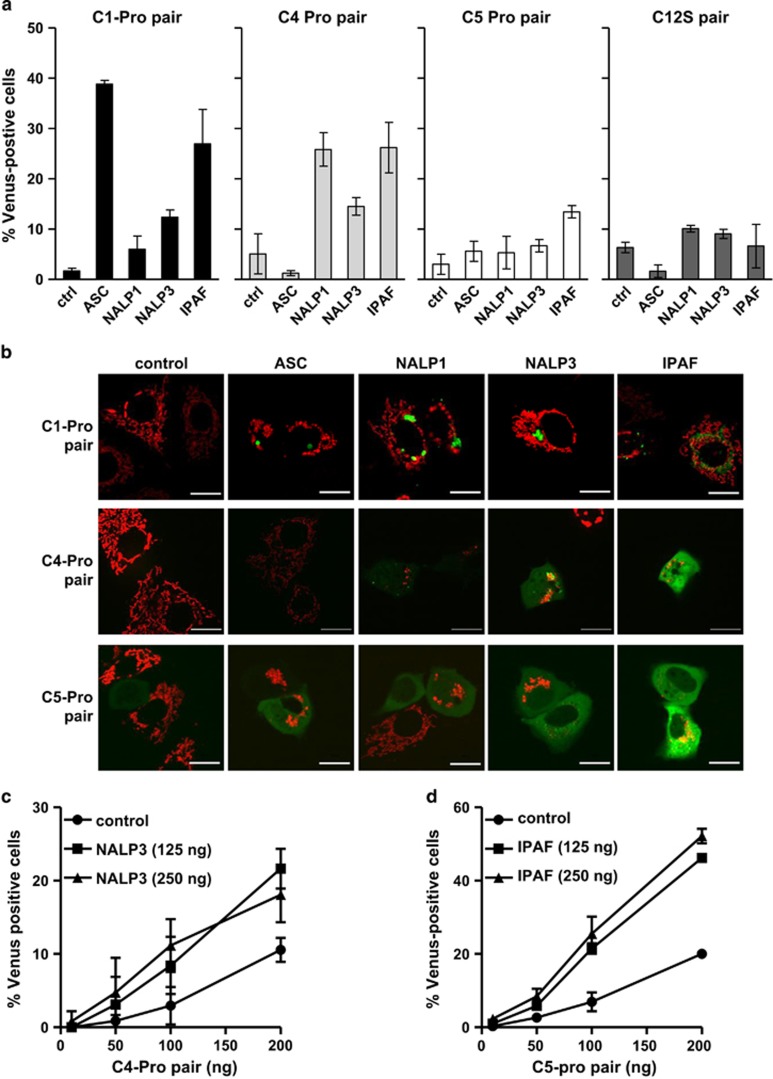
Inflammatory caspase BiFC is induced by distinct inflammasome components. (**a**) MCF-7 cells were transiently transfected with C1-Pro (10 ng of each), C4-Pro (50 ng of each), C5-Pro (200 ng of each) or C12S (20 ng of each) BiFC plasmid pairs along with expression plasmids encoding ASC, NALP1, NALP3 or IPAF as indicated. All wells also received dsRed-mito (10 ng) as a reporter for transfection. All cells were treated with qVD-OPH (5 *μ*M) to prevent cell death. At 48 h after transfection, the percentage of transfected cells that were Venus-positive was determined from a minimum of 300 cells per well. Results represent triplicate counts with error bars representing S.D. and are representative of at least three independent experiments. (**b**) Representative confocal images of cells from (**a**) are shown. Mitochondria are shown in red and caspase BiFC in green. Scale bars represent 20 *μ*m (**c**) MCF-7 cells were transiently transfected with the indicated amounts of C4-Pro VC, C4 Pro VN and NALP3. Cells were treated and assessed at 48 h as in (**a**). (**d**) MCF-7 cells were transiently transfected with the indicated amounts of C5-Pro VC, C5-Pro VN and IPAF. Cells were treated and assessed at 48 h as in (**a**)

Next, we compared the BiFC responses of caspase-1 to a range of inflammasome components (Figure 2a). As before, ASC induced robust C1-Pro BiFC, whereas the expression of NALP1 and NALP3 induced much lower levels of C1-Pro BiFC. In contrast, coexpression of IPAF induced C1-Pro BiFC in over 30% of the cells. NALP1 and NALP3 both require ASC to bind to caspase-1, whereas IPAF directly interacts with caspase-1.^7, 8, 11^ Therefore, the BiFC results for caspase-1 are consistent with the published interactions of these proteins.

Representative images of caspase-1 BiFC demonstrated distinct organizations of the fluorescent complexes induced by the expression of individual inflammasome components (Figure 2b). ASC induced punctate complexes of C1-Pro BiFC with a single punctum per cell. In the few cells where NALP1 and NALP3 induced caspase-1 BiFC, it localized to multiple, larger, more disordered complexes. Finally, IPAF-induced caspase-1 BiFC resulted in a punctate distribution of multiple smaller complexes in the cytosol.

We similarly generated VC and VN epitope-tagged versions of the caspase-4-Pro (C4-Pro; aa 1–88), the caspase-5 prodomain (C5-Pro, aa 1–134) and C12S (aa 1–113). We did not observe a measureable amount of C4-Pro BiFC in response to ASC. However NALP1, NALP3 and IPAF each induced C4-Pro BiFC (Figure 2a). Although the effect was small, NALP3-induced C4-Pro BiFC was robust over a range of C4-Pro BiFC pair plasmid concentrations and NALP3 concentrations (Figure 2c), suggesting that caspase-4 may be recruited to the NALP3 inflammasome, as well as the NALP1 and IPAF inflammasomes. NALP1, NALP3 and IPAF induced a predominantly diffuse, cytosolic pattern of C4-Pro BiFC. We noted the additional presence of some brighter punctate C4-Pro BiFC complexes in response to NALP1 and NALP3 (Figure 2b).

C5-Pro BiFC was most efficiently induced by IPAF (Figure 2a). Again, this result was robust over a range of C5-Pro BiFC pair concentrations and IPAF concentrations (Figure 2d). This suggests that caspase-5 may be recruited to the IPAF inflammasome. C5-Pro BiFC fluorescence was diffuse throughout the cell compared with the more restricted distribution of the IPAF-induced C1-Pro BiFC (Figure 2b). Although ASC, NALP1 and NALP3 induced minimal C5-Pro BiFC, the fluorescence distribution was also diffuse in the few cells that were BiFC-positive.

C12S BiFC was not detected in response to any of the inflammatory proteins tested (Figure 2a). Taken together, these results demonstrate that we can detect BiFC of inflammatory caspases in response to distinct inflammasome components, indicating that the BiFC observed represents dimerization of inflammatory caspases upon recruitment to inflammasomes.

### Caspase-1 forms heterodimers with caspase-4 and caspase-5

**Figure 3:**
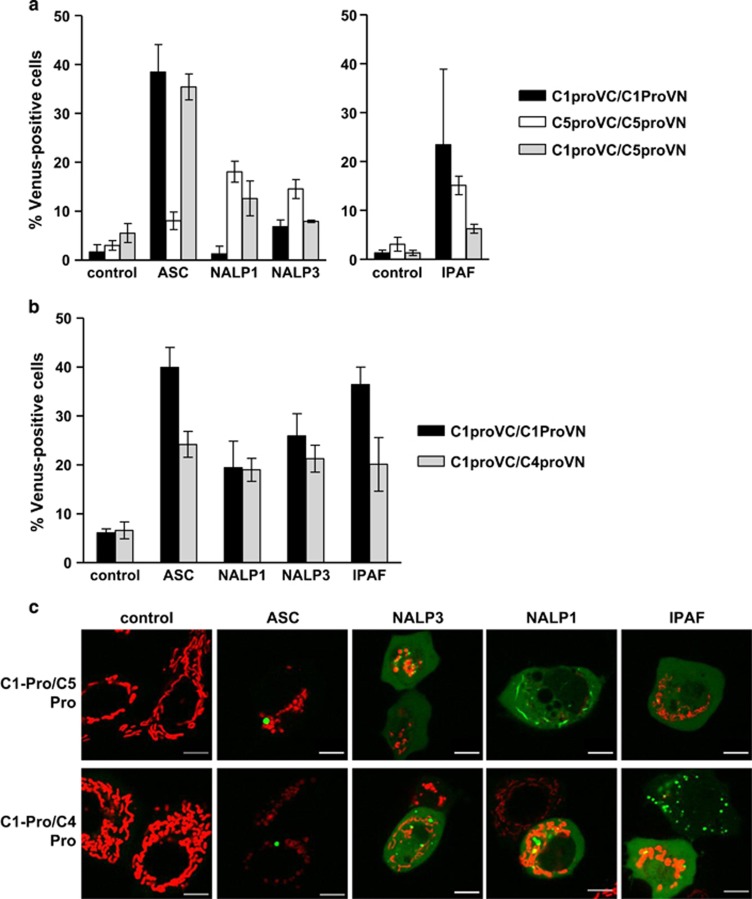
Heterodimerization of caspase-1 with caspase-4 or -5 is induced by inflammasome components. (**a**) MCF-7 cells were transiently transfected with the indicated combinations of C1-Pro (10 ng) and C5-Pro (200 ng) BiFC plasmids along with expression plasmids encoding ASC, NALP1, NALP3 or IPAF. All wells also received dsRed-mito (10 ng) as a reporter for transfection. All cells were treated with qVD-OPH (5 *μ*M) to prevent cell death. At 48 h after transfection, the percentage of transfected cells that were Venus-positive was determined from a minimum of 300 cells per well. Results represent triplicate counts with error bars representing S.D. and are representative of at least three independent experiments. (**b**) MCF-7 cells were transiently transfected with the indicated combinations of C1-Pro (10 ng) and C4-Pro (20 ng) BiFC plasmids along with expression plasmids encoding ASC, NALP1, NALP3 or IPAF and dsRed-mito (10 ng). Cells were treated and assessed at 48 h as in (**a**). (**c**) Representative confocal images of cells from (**a**) and (**b**) are shown, with the mitochondria shown in red and caspase BiFC shown in green. Scale bars represent 10 *μ*m

Caspase-5 has been reported to be recruited to the NALP1 inflammasome in conjunction with caspase-1.^7^ Therefore, we investigated if caspase-1 could form heterodimers with caspase-5. We expressed the C1-Pro BiFC pair, the C5-Pro BiFC pair or a combination of C1-Pro VC and C5-Pro VN with ASC, NALP1, NALP3 or IPAF (Figure 3a). We observed that ASC induced similar levels of paired C1-Pro BiFC and hybrid C1-Pro/C5-Pro BiFC, indicating that these caspases may form heterodimers in response to ASC. NALP1 also induced C1-Pro/C5-Pro BiFC but to a lesser extent compared with that induced by ASC. We similarly explored the ability of caspase-4 to heterodimerize with caspase-1. The combination of C4-Pro and C1-Pro induced robust BiFC in response to ASC, NALP1, NALP3 and IPAF (Figure 3b).

The appearance of the caspase-1/-5 BiFC and the caspase-1/-4 BiFC complexes in response to ASC was very similar to the caspase-1 BiFC complexes, appearing as a single punctum per cell. NALP1, NALP3 and IPAF expression resulted in an intermediate distribution of diffuse and punctate C1-Pro/C4-Pro BiFC fluorescence. NALP1- and NALP3-induced C1-Pro/C5-Pro BiFC resulted in an intermediate distribution of diffuse and punctate spots in the few cells that were positive. IPAF-induced C1-Pro/C5-Pro BiFC was diffuse throughout the cell (Figure 3c).

### Caspase BiFC reveals inflammasome structures in cells

**Figure 4:**
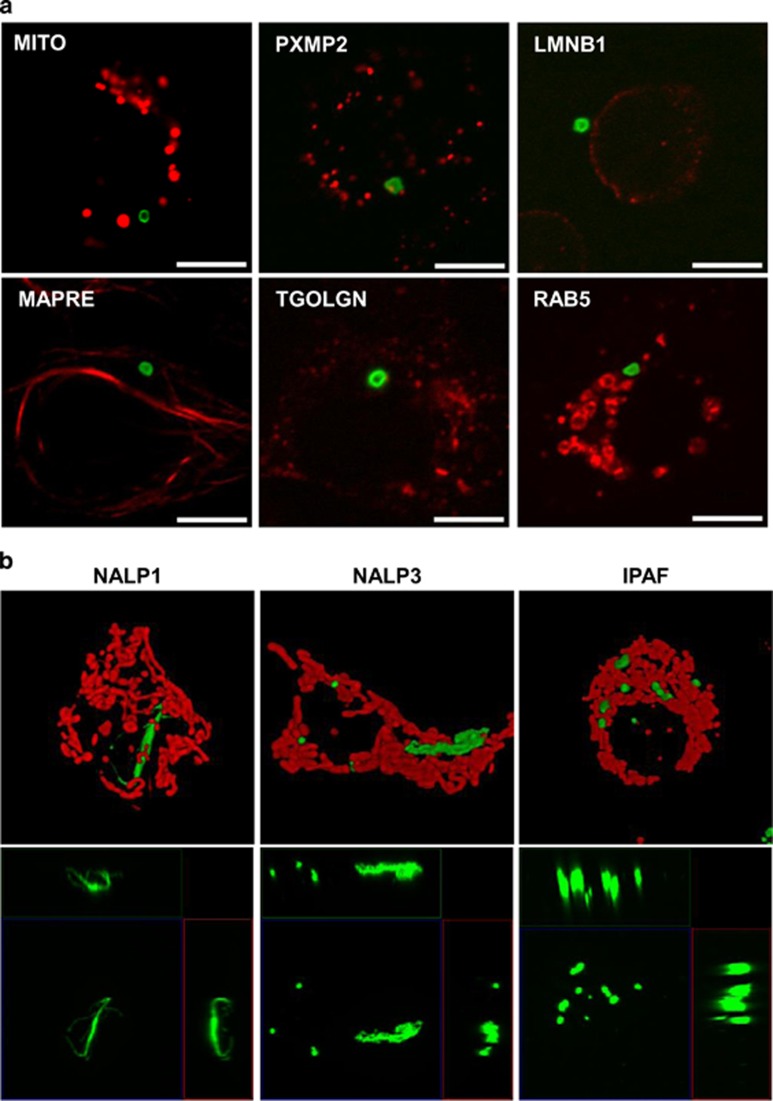
Subcellular localization of C1-Pro BiFC. (**a**) MCF-7 cells were transiently transfected with expression plasmids encoding C1-Pro VC (10 ng) and C1-Pro VN (10 ng) along with an expression plasmid encoding ASC (125 ng) and dsRed-mito, PXMP2-RFP, LNMB1-RFP, MAPRE-RFP, TGOLGN-RFP or Rab5-RFP as indicated. All cells were treated with qVD-OPH (5 *μ*M) to prevent cell death. Cells were assessed for BiFC (green) 24 h after transfection. Representative images are shown, taken with a x64 objective. DsRed-mito, PXMP2-RFP, LNMB1-RFP, MAPRE-RFP, TGOLGN-RFP and Rab5-RFP were used to detect mitochondria, peroxisomes, nuclei, cytoskeleton, Golgi and early endosomes, respectively (red). Scale bars represent 10 *μ*m. (**c**) MCF-7 cells were transiently transfected with expression plasmids encoding C1-Pro VC (10 ng) and C1-Pro VN (10 ng) along with expression plasmids encoding NALP1, NALP3 or IPAF (125 ng) and dsRed-mito (10 ng) in the presence of qVD-OPH (5 *μ*M). Cells were assessed for C1-Pro BiFC (green) 24 h after transfection. Images in the top row are three-dimensional (3D) isosurface rendering reconstructions composed from 0.1 *μ*m serial confocal images through the *z* plane of the cell showing mitochondria in red and C1-Pro BiFC (in green). The bottom row shows the orthogonal views maximum intensity projection of the Venus signal for each condition. The middle panel is the *xy* plane, the right panel is the *yz* plane and the top panel is the *xz* plane

Our results show that ASC-induced C1-Pro BiFC appeared as a single ‘speck' or punctum per cell, which is consistent with published reports of ASC localization.^9, 22^ To further explore these BiFC structures, we imaged ASC-induced C1-Pro BiFC complexes at higher magnifications and resolution. We observed that the specks of C1-Pro BiFC were hollow ring-like structures. The ASC/C1-Pro complexes appeared to be perinuclear and did not colocalize with mitochondria (mito), peroxisomes (peroxisomal membrane protein 2 (PXMP2)), nuclei (lamin B1), cytoskeleton (microtubule-associated protein RP/EB (MAPRE)), Golgi (tGolgn) or early endosomes (Rab5) (Figure 4a and Supplementary Movie S1).

We similarly imaged cells expressing the C1-Pro BiFC pair with NALP1, NALP3 or IPAF. The 3D images of these cells demonstrate that NALP1-, NALP3- and IPAF-induced caspase-1 BiFC formed very distinct complexes compared with those induced by ASC. The C1-Pro BiFC complexes induced by NALP1 and NALP3 were less uniform and formed into filamentous structures that distributed throughout the cell (Figure 4b and Supplementary Movies S2 and S3). In contrast, IPAF-induced C1-Pro BiFC resulted in a series of smaller round complexes (Supplementary Movie S4). The 3D images suggest that the C1-Pro BiFC structures assembled in response to NALP1, NALP3 or IPAF expression were not hollow compared with those induced by ASC expression.

**Figure 5:**
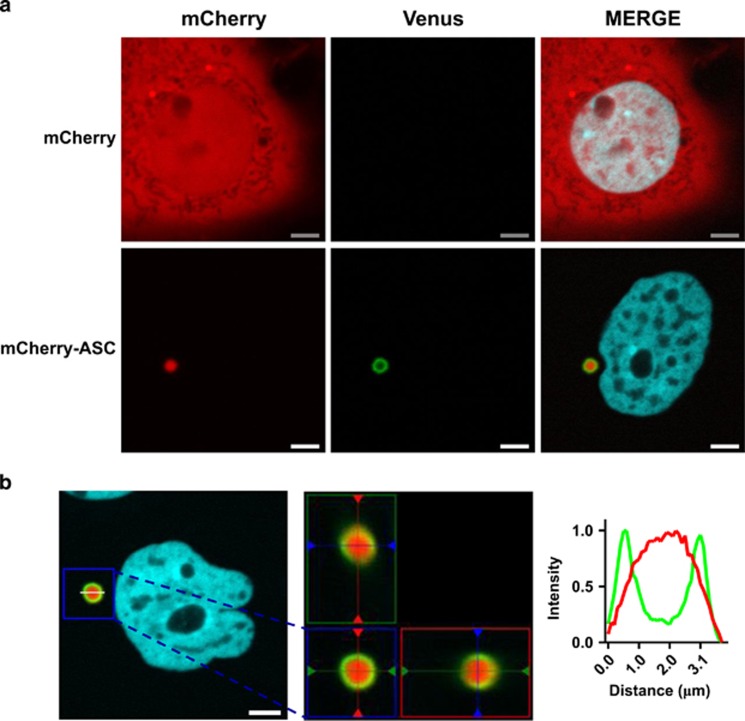
Caspase-1 is dimerized on the outer surface of ASC complexes. MCF-7 cells were transiently transfected with expression plasmids encoding C1-Pro VC (40 ng), C1-Pro VN (40 ng) and mTurquoise-histone H2A-10 (blue), which localizes to the nucleus (10 ng), with mCherry (800 ng) or ASC-mCherry (800 ng) in the presence of qVD-OPH (20 *μ*M). After 24 h, cells were assessed for caspase-1 BiFC (green) and ASC (red) colocalization. Scale bars represent 5 *μ*m. (**b**) 3D reconstructions composed from 0.1 *μ*m serial confocal images through the *z* plane of the cell were made. The orthogonal slice view of the boxed region (left) is shown (center). The middle panel is the *xy* plane, the right panel is the *yz* plane and top panel is the *xz* plane. The *yz* and *xz* planes intersect according to the cross hairs. The line scan (right) indicates the localization of C1-Pro BiFC relative to mCherry-ASC and corresponds to the line drawn on the image (left)

To determine where caspase-1 BiFC localizes in relation to ASC, we coexpressed a fluorescently tagged version of ASC, mCherry-ASC, with the C1-Pro BiFC pair. Expression of mCherry-ASC resulted in a single large fluorescent perinuclear punctum in each cell. C1-Pro BiFC did not colocalize exactly with mCherry-ASC but rather formed a distinct ring around the mCherry-ASC punctum (Figures 5a and b). 3D imaging of these complexes indicated that C1-Pro BiFC completely encapsulated mCherry-ASC (Figure 5b). The C1-Pro BiFC structures induced by mCherry-ASC had an average diameter of 2.5 *μ*m ranging from 1.433  to 4.812 *μ*m. The C1-Pro BiFC spheres induced by ASC had an average diameter of 1.9 *μ*m ranging from 1.229 to 2.662 *μ*m (Supplementary Figure S2). It is likely that the larger average size of the former complexes is because of the presence of the mCherry moiety. Diameters were measured in both the *x* and *y* plane and there was a strong correlation between these values, indicating that the majority of these shapes were spherical in nature. These results suggest that ASC recruits caspase-1 such that caspase-1 is dimerized on the external surface of the spherical ASC complex.

### Inflammatory caspase BiFC is induced by PAMPs in macrophages

**Figure 6:**
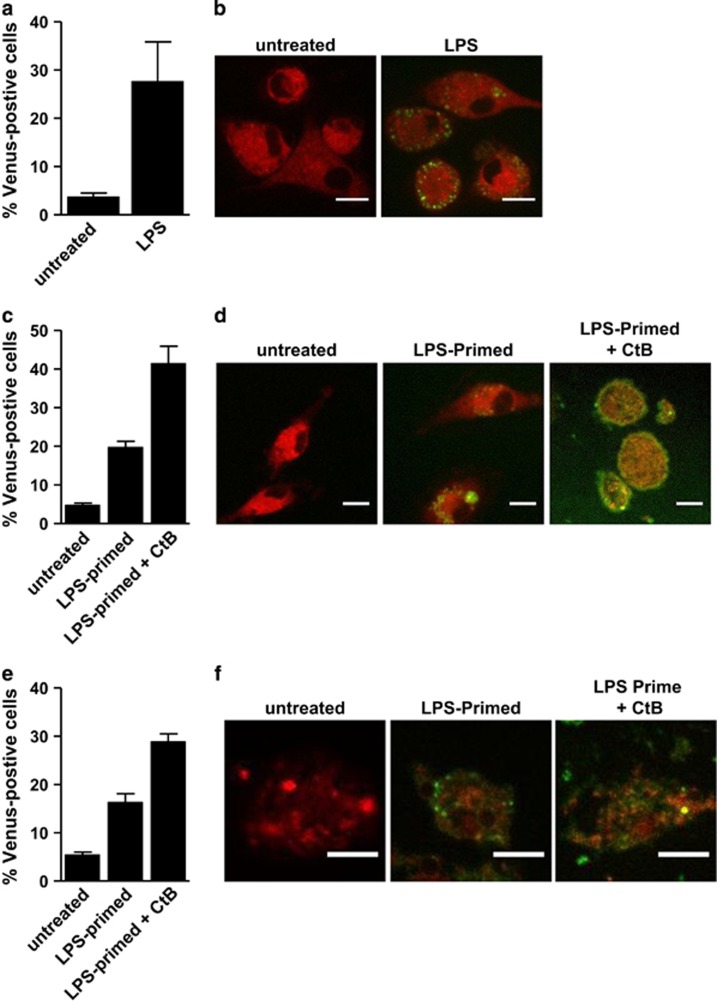
Proinflammatory stimuli induce inflammatory caspase BiFC in primary murine BMDM. (**a**) Murine BMDM were transiently transfected with expression plasmids encoding C1-Pro VC (300 ng) and C1-Pro VN (300 ng) with dsRed as a reporter for transfection. After 24 h, cells were treated with or without ultrapure LPS (100 ng/ml) for 4 h. The percentage of transfected cells that were Venus-positive cells was determined by flow cytometry. Error bars represent the S.D. of three independent experiments. (**b**) Representative confocal images of cells from (**a**) are shown. Scale bars represent 10 *μ*m. (**c**) Murine BMDM were transiently transfected with expression plasmids encoding C5-Pro VC (300 ng) and C5-Pro VN (300 ng) with dsRed as a reporter for transfection. After 24 h, cells were left untreated or primed with ultrapure LPS (100 ng/ml) for 4 h. LPS-primed cells were then treated with CtB (20 *μ*g/ml) for 16 h. The percentage of transfected cells that were Venus-positive cells was determined by flow cytometry. Error bars represent the S.D. of three independent experiments. (**d**) Representative confocal images of cells from (**c**) are shown. Scale bars represent 10 *μ*m. (**e**) Murine BMDM were transiently transfected with expression plasmids encoding C1-Pro VC (300 ng) and C5-Pro VN (300 ng) with dsRed as a reporter for transfection. Cells were treated and assessed as in (**c**). (**f**) Representative confocal images of cells from (**e**) are shown. Scale bars represent 50 *μ*m

We next explored if BiFC could detect inflammatory caspase dimerization associated with the engagement of inflammasome receptors by their natural ligands. Primary murine bone marrow-derived macrophages (BMDM) were transiently transfected with the C1-Pro BiFC components followed by treatment with ultrapure LPS. Treatment with LPS for 4 h resulted in an increase in the number of BiFC-positive cells over background (Figure 6a). LPS treatment of BMDM induced robust IL-1*β* release in a caspase-dependent manner (Supplementary Figure S3). The release of IL-1*β* was increased by further treatment with ATP, which activates the NALP3 inflammasome. However, ATP also induced massive death of the cells preventing the analysis of any further induction of C1-Pro BiFC. LPS induced a punctate pattern of C1-Pro BiFC, which was similar to that induced by the overexpression of ASC (Figure 6b). In contrast to overexpression of ASC, however, LPS induced multiple puncta per cell rather than one large complex.

To determine if we could detect dimerization of other inflammatory caspases in primary BMDM in response to pro-inflammatory stimuli, we performed similar experiments with the caspase-5-Pro BiFC pair. Priming of BMDM with LPS for 4 h also induced caspase-5 BiFC to a level that was comparable to that of caspase-1, with a similar subcellular distribution (Figure 6c). Cholera toxin subunit B (CtB) has been shown to activate caspase-11, the murine homolog of caspase-5, in LPS-primed macrophages.^16^ Therefore, we investigated the ability of CtB to induce caspase-5 dimerization, as measured by BiFC. Strikingly, further treatment of LPS-primed BMDM with CtB substantially increased the level of caspase-5 dimerization. The localization of fluorescence representing caspase-5 dimerization became diffuse throughout the cell with an accumulation at the cell membrane in many cells (Figure 6d).

We assessed if we could detect heterodimerization of caspase-1 with caspase-5 in primary macrophages. BMDM were transfected with C1-Pro VC and C5-Pro VN. Treatment of these cells with LPS for 4 h induced Venus-positive cell numbers that were robustly increased by subsequent treatment with CtB (Figure 6e). In comparison with CtB-induced caspase-5 homodimers, the caspase-1/caspase-5 BiFC appeared as puncta in the cell (Figure 6f).

## Discussion

We have previously used the caspase BiFC approach to interrogate the caspase-2 pathway and reported that it was a robust and specific method to measure induced proximity of caspase-2 upon recruitment to its activation platform, which is the most apical step in the activation of caspase-2.^19^ In this current study, we have extended the technique to the human inflammatory caspases, caspase-1, -4, -5 and -12, revealing a number of novel interactions at the level of the inflammasome.

Although the role of caspase-1 in cytokine processing and inflammation is well understood, the contributions of the remaining inflammatory caspases, caspase-4, -5 and -12, are unclear. Despite a high degree of peptide substrate specificity between caspase-1, -4 and -5,^23^ caspase-4 and -5 have a more limited substrate repertoire than caspase-1. Neither of them appears to cleave IL-1*β*.^24^ In addition, a reverse degradomics approach revealed only 3 putative caspase-4 substrates and 0 caspase-5 substrates compared with 55 putative caspase-1 substrates from more than 500 identified N termini.^25^ The absence of a defined physiological substrate for caspase-4 or -5 has made it difficult to determine the precise contribution of these proteins to inflammatory pathways.

Caspase-4 and -5 have been described as intracellular LPS receptors. Electroporation of ultrapure LPS into human monocytes and keratinocytes induced pyroptosis in a caspase-4-dependent but ASC-independent manner. This appears to be because of the ability of LPS to bind caspase-4 (and caspase-5), inducing oligomerization of the caspase.^17^ The results of that study suggest that this function of caspase-4 and -5 is independent of other inflammasome components. However, our study reveals that caspase-4 and -5 can be recruited to inflammasome complexes under certain circumstances. Caspase-4 dimerization was induced by NALP1, NALP3 and IPAF overexpression, whereas caspase-5 dimerization was induced by IPAF. Heterodimerization of caspase-1 with -5 was detected in response to ASC and NALP1, and heterodimerization of caspase-1 with -4 was induced by a range of inflammasome proteins. Finally, caspase-5 dimerization and caspase-1/-5 heterodimerization was induced by LPS and by CtB in LPS-primed cells, which may be an evidence for activation of the noncanonical pathway analogous to murine caspase-11 activation.^16^ Whether the latter dimerization events are dependent on specific inflammasome proteins is to be determined. Taken together, these results suggest that caspase-4 and -5 have direct roles in inflammasome assembly and function.

The heterodimerization of caspase-1 with caspase-4 or -5 detected using BiFC suggests an alternative mechanism of regulating caspase-1 activation. Our results are consistent with the first biochemical analysis of the NALP1 inflammasome that showed caspase-5 as a component of the complex.^7^ Only the components of this inflammasome, NALP1 and ASC induced detectable caspase-1/-5 heterodimerization. Thus, the formation of caspase-1/-5 heterodimers may result from the recruitment of both caspases to the NALP1 inflammasome. However, it is unclear what the functional consequences of such heterodimers are. Caspase-11, the murine ortholog of caspase-4 and -5, is required for caspase-1 activation in certain circumstances.^16^ In addition, caspase-4 transgenic mice show evidence of increased caspase-1 activity and increased sensitivity to LPS.^26^ These events may be because of heterodimerization of caspase-1 with caspase-4 or -11. Thus, heterodimerization of caspase-1 with caspase-4 or -5 may represent a means to activate or enhance activation of caspase-1. Alternatively, heterodimers of caspase-1 with caspase-5 or -4 could result in a distinct enzymatic complex. These may function in an analogous manner to heterodimers formed between caspase-8 and the long isoform of cellular FLICE inhibitory protein (FLIP_L_). Although caspase-8/FLIP_L_ heterodimers can activate caspase-8, the resulting complex has a more limited substrate repertoire and exerts prosurvival functions rather than death.^27^ Thus, caspase-1/-5 or -1/-4 heterodimer complexes may function distinctly from caspase-1 homodimers in the regulation of cytokine processing and cell death.

The differences in the organization and distribution of BiFC structures associated with heterodimerization or homodimerization of inflammatory caspases may also indicate different functions. For example, caspase-5 BiFC always appeared as diffuse throughout the cytosol, whereas caspase-1 BiFC formed distinct structures in response to coexpression of different inflammasome proteins. Caspase-1/-5 BiFC and caspase-1/-4 BiFC organized into an intermediate pattern of punctate and diffuse fluorescence in response to NALP1 and other inflammasome proteins but appeared as a single punctum in response to ASC. These varied organizations of the BiFC structures may be related to functional differences in the resulting inflammasome complexes. For example, the different distributions of the dimerized caspases may restrict access to distinct subsets of substrates. In macrophages, the different distributions of caspase-5 BiFC in LPS-primed cells (punctate in the cytoplasm) and CtB-treated cells (plasma membrane bound) suggest that two separate responses of caspase-5 may exist. Specifically, the inflammasomes may be assembled in the cytosol in response to LPS or at the plasma membrane in response to CtB. Taken together, these results indicate that the localization of and distribution of inflammasome assembly is governed by the caspases that are recruited in addition to the specific upstream trigger.

The organization of the caspase-1 BiFC structures in response to ASC expression is consistent with published reports.^22, 28^ Endogenous ASC induced with retinoic acid forms perinuclear specks, and LPS induced similar structures in cells stably expressing GFP-ASC.^9, 22^ Cryo-electron microscopy (EM) of *in vitro* reconstituted AIM2 and NALP3 inflammasomes showed that ASC forms filaments through the PYD as a result of prion-like ASC polymerization.^29^ The resulting filaments are thought to induce CARD clustering, leading to caspase-1 recruitment and the formation of caspase-1 filaments through CARD–CARD interactions. This forms a star-shaped ternary complex with ASC in the center. In the same study, immunogold labeling and EM of ASC-eGFP expression in cells revealed that ASC forms specks that are around 2 μm in size. These appear as dense structures proposed to have a ball-of-yarn-like architecture.^29^ The authors postulated that the ASC specks are comprised of the filamentous structures revealed by cryo-EM. The caspase-1 BiFC spheres we detected ranged from ~1.2 to 2.7 *μ*m in diameter. The addition of a mCherry tag to the ASC protein generally resulted in larger caspase-1 BiFC spheres (up to 4.8 *μ*m) but the organization and localization of the spheres was unchanged. Thus, the sizes and shapes of the caspase-1/ASC structures detected by BiFC are consistent with the previous results obtained using EM. Our data is therefore in line with the model where ASC forms dense spherical structures. Moreover, it clearly demonstrates that dimerized caspase-1 was located on the surface of this complex rather than intertwined with ASC, placing ASC at the center of a giant spherical cluster that recruits caspase-1 molecules to its surface.

Inflammasome signaling underlies the pathophysiology of a number of infectious and sterile inflammatory conditions. Mutations in NALP3 are associated with hereditary autoinflammatory conditions, including Muckle–Wells syndrome and familial cold autoinflammatory syndrome.^30, 31^ Using BiFC to measure caspase-1 dimerization has allowed us to identify the conditions under which caspase-1 is recruited to different inflammasomes. This approach can be used to further dissect how caspase-1 is activated during inflammation and how its regulation is compromised in autoinflammatory disorders. Furthermore, the conclusions of our work support a model where the inflammatory caspases, caspase-4 and -5, have key roles in inflammasome regulation.

## Materials and Methods

### Plasmids

All caspase BiFC plasmids were cloned by standard PCR strategies with primers designed to incorporate appropriate restriction enzyme sites, from a full-length caspase-1 (ICE), -4 (ICErel_II_), -5 (ICErel_III_) or -12 cDNA into the pBiFC.VC155 and pBiFC.VN173 plasmids (a gift from Chang-Deng Hu, Purdue University, West Lafayette, IN, USA). Single mutations were introduced using QuikChange Site-Directed Mutagenesis Kit (Agilent Technologies, Santa Clara, CA, USA). After cloning, each construct was verified by sequencing. Expression plasmids for ASC and NALP1 were a gift from Seamus Martin (Trinity College, Dublin, Ireland) and expression plasmids encoding IPAF and NALP3 were purchased from Open Biosystems (GE Dharmacon, Lafayette, CO, USA). mTurquoise-H2A-10 was a gift from Michael Davidson (Addgene, Cambridge, MA, USA; plasmid no. 55556). Human caspase-12 cDNA was a gift from Maya Salah (McGill University, Montreal, QC, Canada).

### Transient transfection assays

In total, 1 × 10^5^ HeLa or MCF-7 cells were transfected with appropriate plasmid combinations using Lipofectamine 2000 transfection reagent (Invitrogen, Grand Island, NY, USA) according to the manufacturer's instructions. Murine BMDM were transfected using Lipofectamine LTX with Plus™ transfection reagent (Invitrogen) according to the manufacturer's instructions. Cells were transfected with amounts of the relevant expression plasmids as described in the figure legends.

### Microscopy

Cells were imaged using a spinning disk confocal microscope (Zeiss, Thornwood, NY, USA), equipped with a CSU-X1A 5000 spinning disk unit (Yokogowa Electric Corporation, Tokyo, Japan), multilaser module with wavelengths of 458, 488, 514 and 561 nm, and an Axio Observer Z1 motorized inverted microscope equipped with a precision motorized XY stage (Carl Zeiss MicroImaging, Thornwood, NY, USA). Images were acquired with a Zeiss Plan-Neofluar 40 × 1.3 NA or 64 × 1.4 NA objective on an Orca R2 CCD camera using Zen 2012 software (Zeiss, Thornwood, NY, USA). Cells were plated on dishes containing coverslips coated with fibronectin (Mattek Corp., Ashland, MA, USA) 24 h before transfection.

### Bone marrow-derived macrophages

BMDM were generated from bone marrow progenitors obtained from C57BL/6J mice. Freshly prepared bone marrow cells were cultured in DMEM medium supplemented with 10% heat-inactivated FCS, 2 mM l-glutamine, 10 mM HEPES, 50 μg/ml penicillin and non-essential amino acids in the presence of 20 ng/ml rmM-CSF (Peprotech, Rocky Hill, NJ, USA) for 6 days. Unattached cells were removed on day 6, and attached macrophages were detached from plates and replated for experimental use.

### Flow cytometry

Cells were treated with Ultrapure LPS (from *Escherichia coli* O111:B4; Invivogen, San Diego, CA, USA) or CtB (List Biological Laboratories, Campbell, CA, USA) as indicated and collected by cell scraping followed by centrifugation. DsRed- and Venus-positive cells were quantified by flow cytometry and analyzed with the FlowJo software (FlowJo LLC, Ashland, OR, USA).

### Immunoblotting

Cell lysates were made by resuspending cells in three cell pellet volumes of RIPA buffer (50 mM Tris-Cl, pH 7.4,150 mM NaCl, 2 mM EDTA, 0.5% sodium deoxycholate, 1% Nonidet P-40 and 0.1% SDS) containing protease inhibitors (Complete Mini Protease Inhibitor Cocktail; Roche, Indianapolis, IN, USA). Lysates were resolved by SDS-PAGE. The proteins were transferred to PVDF (Bio-Rad, Hercules, CA, USA) and immunodetected using appropriate primary and peroxidase-coupled secondary antibodies (GE Healthcare Life Sciences, Pittsburg, PA, USA). Proteins were visualized by West Pico and West Dura Chemiluminescence Substrate (Pierce, Rockford, IL, USA).

### ELISA IL-1*β* measurements

IL-1*β* levels in harvested supernatants were evaluated with the Mouse IL-1*β* Quantikine ELISA Kit (R&D Systems, Minneapolis, MN, USA) according to the manufacturer's instructions.

## Abbreviations

AIM2: absent in melanoma 2
ASC: apoptosis-associated speck-like protein containing a CARD
ATP: adenosine triphosphate
BiFC: bimolecular fluorescence complementation
BMDM: bone marrow-derived macrophage
CARD: caspase recruitment domain
CtB: cholera toxin subunit B
EM: electron microscopy
FLIP_L_: long isoform of cellular FLICE inhibitory protein
IL: interleukin
IPAF: ICE protease-activating factor
LPS: lipopolysaccharide
MAPRE: microtubule-associated protein RP/EB
NALP: NACHT leucine-rich repeat protein
NLRC4: NLR family CARD domain-containing protein 4
NLRP: NOD-like receptor protein
PAMP: pathogen-associated molecular pattern
PXMP2: peroxisomal membrane protein 2
PYD: pyrin domain
VC: Venus C
VN: Venus N
