# Supplementary material for: Single-cell imaging of inflammatory caspase dimerization reveals differential recruitment to inflammasomes
Source: Cell Death Dis. 2015 Jul 9;6(7):e1813–. doi: 10.1038/cddis.2015.186 (PMC4650733; doi:10.1038/cddis.2015.186)
Supplement: Supplementary Figures [file cddis2015186x1.pdf]

Supplemental Figure S1

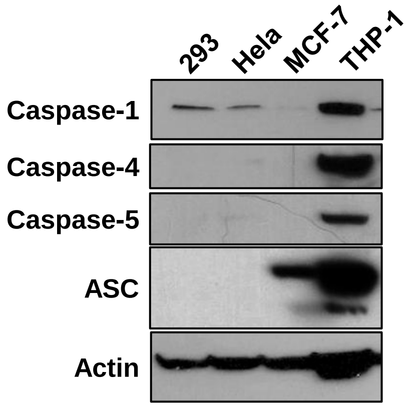

Supplemental Figure 1: Inflammatory caspase expression in cell lines
Total protein lysates were prepared from the indicated cell lines and assessed for caspase-1, -4, -5, or ASC expression by immunoblot. Blots were probed for actin as a loading control.

Supplemental Figure S2

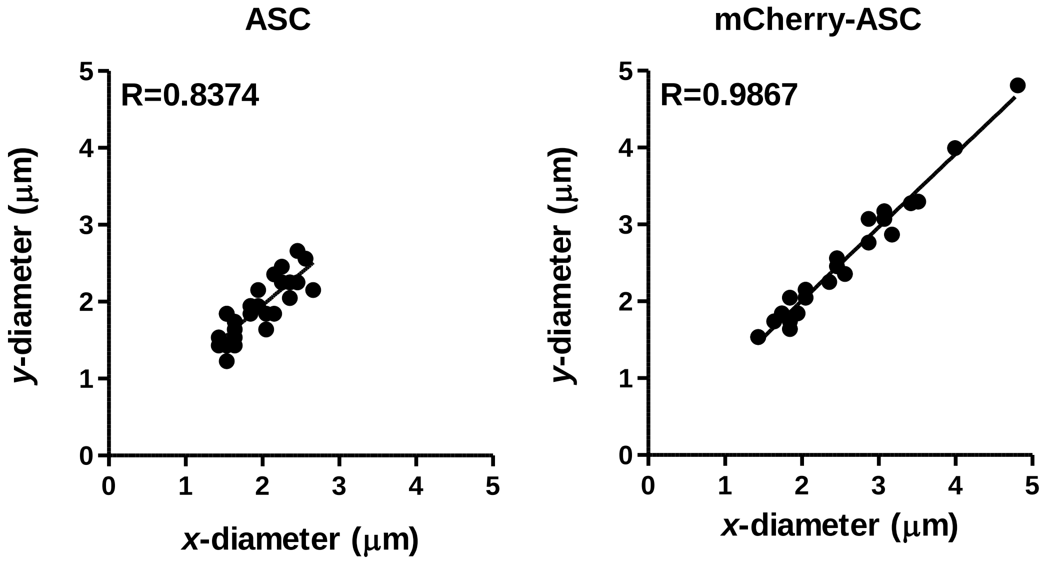

Supplemental Figure 2: Diameter of ASC-induced caspase-1 BiFC structures
The diameters of caspase-1 BiFC structures induced by ASC, as shown in Figure 4A (left), and by mCherry-ASC, as shown in Figure 5 (right), were measured. Measurements were made in *x* and *y* in the orthogonal slices view at the *z* plane where the structure was widest. Each point on the graph represents an individual ASC/caspase-1 punctum, n=28 for ASC and n=23 for mCherry ASC. The line represents the linear regression and R is the Pearson coefficient.

Supplemental Figure S3

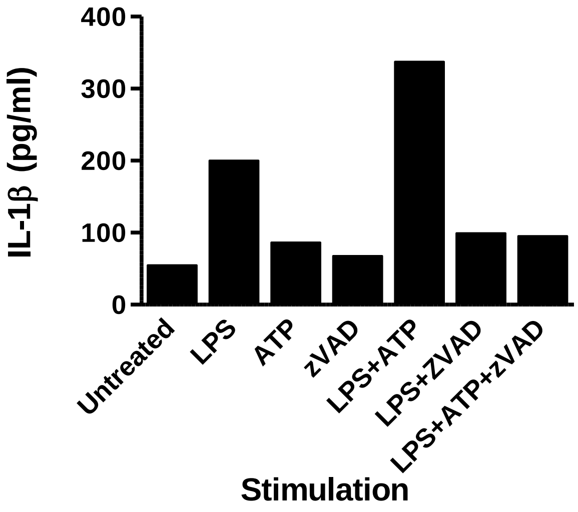

Supplemental Figure 3: LPS induces IL-1β release in murine bone marrow derived macrophages (BMDM)

Primary BMDM were treated with ultrapure LPS (0.1 µg/ml) for 18 h, ATP (5 mM) for 1 h or zVAD (10 µM). Cell culture supernatants were collected and the levels of IL-1β were measured by ELISA. Results represent triplicate measurements with error bars representing standard deviation. Standard deviation was less than 0.09 for all treatments and therefore the error bars are not visible on the graph
