# Supplementary material for: Single-cell imaging of inflammatory caspase dimerization reveals differential recruitment to inflammasomes
Source: Cell Death Dis. 2015 Jul 9;6(7):e1813–. doi: 10.1038/cddis.2015.186 (PMC4650733; doi:10.1038/cddis.2015.186)
Supplement: Supplementary Information [file cddis2015186x2.doc]

**Supplemental Material**

*Supplemental Figures 1-3*

*Supplemental movies:*

**Movie S1.** **ASC-induced caspase-1-Pro BiFC localizes to large peri-nuclear spherical complexes.** 3D isosurface rendering reconstruction, rotated around the *yz*-axis of confocal images through the *x*-plane of an MCF-7 cell expressing ASC, the C1-Pro BiFC pair (*green*) and LNMB1-RFP (*red*).

**Movie S2.** **NALP1-induced caspase-1-Pro BiFC localizes to large filamentous complexes.** 3D isosurface rendering reconstruction, rotated around the *yz*-axis of confocal images through the *x*-plane of an MCF-7 cell expressing NALP1, the C1-Pro BiFC pair (*green*) and dsRed-mito (*red*).

**Movie S3.** **NALP3-induced caspase-1-Pro BiFC localizes to large filamentous complexes.** 3D isosurface rendering reconstruction, rotated around the *yz*-axis of confocal images through the *x*-plane of an MCF-7 cell expressing NALP3, the C1-Pro BiFC pair (*green*) and dsRed-mito (*red*).

**Movie S4.** **IPAF-induced caspase-1-Pro BiFC localizes to multiple small punctate complexes.** 3D isosurface rendering reconstruction, rotated around the *yz*-axis of confocal images through the *x*-plane of an MCF-7 cell expressing IPAF, the C1-Pro BiFC pair (*green*) and dsRed-mito (*red*).
